# Descriptive statistics and regressions of 2D:4D and educational attainment based on RLMS data

**DOI:** 10.1016/j.dib.2017.04.009

**Published:** 2017-04-28

**Authors:** John V.C. Nye, Maksym Bryukhanov, Sergiy Polyachenko

**Affiliations:** a George Mason University, Carow Hall 1D3, Fairfax, VA 22030, USA; b National Research University – Higher School of Economics, Moscow

**Keywords:** RLMS, Descriptive statics, Generalized ordered logit

## Abstract

We document the descriptive statistics and detailed regression outputs for educational attainment and measured 2D:4D ratios, based on the RLMS data (20th round, conducted in 2011–2012). Regression analysis is conducted using STATA 13, gologit2 which is a special code for the generalized ordered logit regression in STATA environment. We provide graphs of differences in means of 2D:4D ratios by educational attainment. Information about the distribution of self-identified nationalities and fields of university degrees of respondents is presented.

Specifications Table

**Table:** 

Subject area	*Economics and human biology*
More specific subject area	*Studies of 2D:4D and behavioral patterns*
Type of data	*Individual survey*
How data was acquired	*Face-to-face interview conducted by ZAO “Demoscope”*
Data format	*SPSS*
Experimental factors	*N/A*
Experimental features	*N/A*
Data source location	http://www.cpc.unc.edu/projects/rlms-hse/project
Data accessibility	*Restricted to Demoscope authorized users*
Related research article	*2D:4D and Lifetime Educational Outcomes: Evidence From The Russian RLMS Survey*

**Value of the data**.

• Descriptive statistics of national self-identities, major fields, educational attainment and fingers’ 2D:4D measurements are helpful in the case of analyzing possible associations between education and 2D:4D.
• Detailed generalized ordered logit regressions’ outputs are helpful in understanding of the general patterns of 2D:4D associations at different levels of education obtained. Inclusion of classical predictors (education of parents) is helpful in foreseeing their correlations with different levels of educational attainment in the studied samples.
• Graphs of means are helpful in comparison of differences between averaged 2D:4D of individuals with university degree and averaged 2D:4D of individuals who completed lower levels of education.

## 1 Experimental design, materials and methods

Measurements were taken by a special team of 2 trained assistants, while other information was taken from the RLMS survey which contains questions regarding an individual׳s socioeconomic characteristics and family background [1]. The data were anonymized before being provided to the authors for statistical analysis. The finger measurements were taken using electronic calipers. Actual measurements were made from the palmar digital crease to the fingertip of the index and ring fingers. Then measurements were rounded to the nearest millimeter.

## 2 Data

**Fig. 1:**
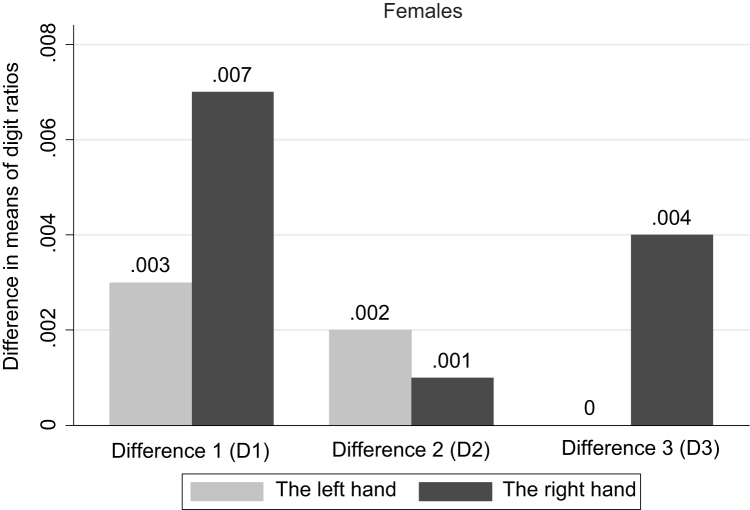
Difference in means of digit ratios by education completed. Females. *Note*: Significant differences are found for the right hand: D1 (at 5% sig. level), D3 (at 10%).

**Fig. 2:**
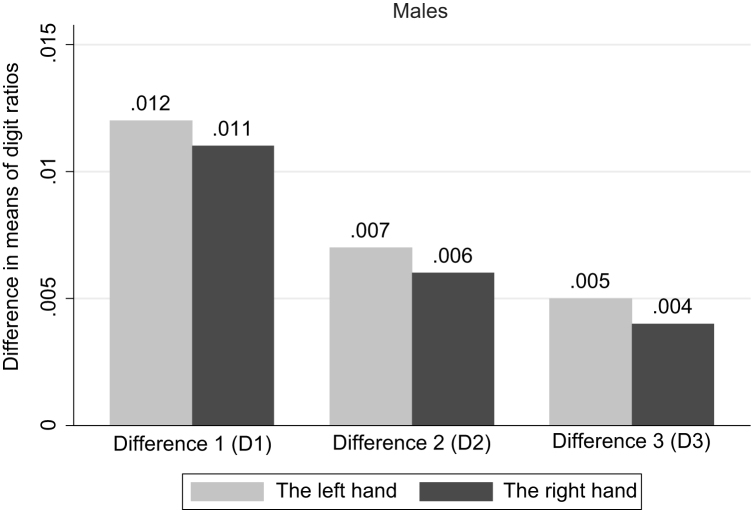
Difference in means of digit ratios by education completed. Males. *Note*: Significant differences are found for: the right hand D1 (at 1% sig. level), D2 (at 5%); the left hand D1 (at 1%), D2 (at 5%).

See Fig. 1, Fig. 2 and Table 1, Table 2, Table 3, Table 4, Table 5, Table 6, Table 7, Table 8, Table 9, Table 10, Table 11, Table 12, Table 13, Table 14, Table 15, Table 16, Table 17.

## Figures and Tables

**Table 1:** Descriptive statistics. Females.

	Mean	Standard deviation	Minimum	Maximum	Number of observations
Digit ratios of the left hand (DL)	0.9988	0.0475	1	1	2148
Digit ratios of the right hand (DR)	0.9991	0.0473	1	1	2146
Educational attainment	3.0083	1.0234	1	4	2172
Uncompleted secondary school education	0.0999	0.2999	0	1	2172
Completed secondary school education	0.2196	0.4141	0	1	2172
Completed vocational school, professional education	0.2528	0.4347	0	1	2172
Completed university degree or higher academic degrees	0.4277	0.4949	0	1	2172
Age	51.1137	17.2483	25	98	2181
Higher education of father	0.2861	0.4521	0	1	1335
Higher education of mother	0.2553	0.4362	0	1	1465
Settlement type 1	0.5854	0.4928	0	1	2132
Settlement type 2	0.0333	0.1795	0	1	2132
Settlement type 3	0.0727	0.2597	0	1	2132
Settlement type 4	0.0647	0.2461	0	1	2132
Settlement type 5	0.0990	0.2987	0	1	2132
settlement type 6	0.1449	0.3521	0	1	2132

*Note*: completed university degree or higher academic degrees refers to the original category “completed higher education and higher levels of education” (the variable “p_diplom”, please, consult: https://www.hse.ru/data/2016/06/07/1247611077/R20_ind_codebook.pdf). Uncompleted secondary school education refers to the category which includes 3 original RLMS categories of educational attainment: 1) 0–6 grades of school; 2) 7–8 grades of school; 3) 7–8 grades of school and some additional education.

**Table 2:** Descriptive statistics. Males.

	Mean	Standard deviation	Minimum	Maximum	Number of observations
Digit ratios of the left hand (DL)	0.9951	0.0456	1	1	1340
Digit ratios of the right hand (DR)	0.9957	0.0461	1	1	1341
Educational attainment	2.8627	1.0521	1	4	1377
Uncompleted secondary school education	0.1082	0.3108	0	1	1377
Completed secondary school education	0.3065	0.4612	0	1	1377
Completed vocational school, professional education	0.1997	0.3999	0	1	1377
Completed university degree or higher academic degrees	0.3856	0.4869	0	1	1377
Age	46.5477	15.9116	25	92	1384
Higher education of father	0.2841	0.4512	0	1	947
Higher education of mother	0.2437	0.4295	0	1	1038
Settlement type 1	0.5916	0.4917	0	1	1354
Settlement type 2	0.0421	0.2009	0	1	1354
Settlement type 3	0.0761	0.2652	0	1	1354
Settlement type 4	0.0569	0.2317	0	1	1354
Settlement type 5	0.1078	0.3103	0	1	1354
settlement type 6	0.1256	0.3315	0	1	1354

*Note*: completed university degree or higher academic degrees refers to the original category “completed higher education and higher levels of education” (the variable “p_diplom”,please, consult: https://www.hse.ru/data/2016/06/07/1247611077/R20_ind_codebook.pdf). Uncompleted secondary school education refers to the category which includes 3 original RLMS categories of educational attainment: 1) 0–6 grades of school; 2) 7–8 grades of school; 3) 7–8 grades of school and some additional education.

**Table 3:** Most frequent fields of university degrees.

Field of a diploma of higher education	ISCO88 Code	Freq.	Percent
Economist	2441	170	12.03
Architects, engineers and related professionals not elsewhere classified	2149	151	10.69
Other teaching professional	2359	126	8.92
Lawyers	2421	92	6.51
Electronics and telecommunications engineers	2144	71	5.02
Mechanical engineers	2145	63	4.46
Architects, town and traffic planners	2141	52	3.68
Medical doctors	2221	51	3.61
Finance and sales associate professionals not elsewhere classified	3419	45	3.18
Electrical engineers	2143	44	3.11

**Table 4:** Generalized logistic regression output, regression coefficients. Dependent variable – educational attainment. Females. The full sample.

	(1)	(2)	(3)	(4)	(5)	(6)	(7)	(8)	(9)	(10)
The first panel										
Digit ratios of the left hand (DL)	3.4843**	0.5971	0.7798	0.7109	0.8881					
	(1.4489)	(1.4371)	(2.4018)	(2.4185)	(2.4144)					
Age		−0.0497***	0.0057	0.0084	0.0107		−0.0495***	0.0055	0.0080	0.0103
		(0.0041)	(0.0140)	(0.0147)	(0.0145)		(0.0041)	(0.0142)	(0.0148)	(0.0147)
Education of father			0.9779**	0.1107	0.0500			0.9797**	0.1100	0.0598
			(0.3803)	(0.4285)	(0.4640)			(0.3817)	(0.4269)	(0.4593)
Education of mother				1.9392***	2.3208***				1.9347***	2.3020***
				(0.6692)	(0.8317)				(0.6671)	(0.8288)
Digit ratios of the right hand (DR)						3.2494**	0.5382	−0.4299	−0.8660	−0.7822
						(1.5158)	(1.2744)	(2.7381)	(2.8042)	(2.8257)
Constant	−1.2660	4.4401***	1.8675	1.7615	1.6130	−1.0337	4.4829***	3.0888	3.3617	3.3018
	(1.4390)	(1.5087)	(2.6610)	(2.6982)	(2.6671)	(1.5055)	(1.3430)	(3.0086)	(3.0760)	(3.0723)

The second panel										
Digit ratios of the left hand (DL)	1.0525	0.6725	−0.4981	−0.2664	−0.5845					
	(0.9940)	(0.9941)	(1.3581)	(1.3774)	(1.4146)					
Age		−0.0130***	−0.0065	−0.0019	0.0011		−0.0136***	−0.0067	−0.0021	0.0010
		(0.0030)	(0.0058)	(0.0059)	(0.0062)		(0.0030)	(0.0058)	(0.0059)	(0.0062)
Education of father			1.3639***	0.6999***	0.6444***			1.3601***	0.6896***	0.6326***
			(0.1730)	(0.2108)	(0.2231)			(0.1732)	(0.2098)	(0.2219)
Education of mother				1.3069***	1.2932***				1.3122***	1.2954***
				(0.2437)	(0.2614)				(0.2434)	(0.2613)
Digit ratios of the right hand (DR)						−0.0037	−0.5748	−1.0692	−1.0191	−1.2166
						(1.0388)	(0.9988)	(1.3277)	(1.3606)	(1.3904)
Constant	−0.2959	0.7538	1.3981	0.8843	1.1687	0.7594	2.0297**	1.9796	1.6482	1.8137
	(0.9928)	(1.0186)	(1.3974)	(1.4182)	(1.4567)	(1.0388)	(1.0328)	(1.3727)	(1.4045)	(1.4323)

The third panel										
Digit ratios of the left hand (DL)	0.6760	0.1289	−0.9949	−0.8382	−0.9564					
	(0.9482)	(0.9617)	(1.3197)	(1.3291)	(1.3709)					
Age		−0.0254***	−0.0405***	−0.0384***	−0.0371***		−0.0254***	−0.0406***	−0.0386***	−0.0372***
		(0.0028)	(0.0055)	(0.0055)	(0.0057)		(0.0029)	(0.0055)	(0.0055)	(0.0057)
Education of father			1.4852***	0.9813***	0.9204***			1.4883***	0.9841***	0.9224***
			(0.1390)	(0.1602)	(0.1621)			(0.1392)	(0.1603)	(0.1619)
Education of mother				1.0046***	0.9620***				1.0048***	0.9619***
				(0.1720)	(0.1762)				(0.1717)	(0.1759)
Digit ratios of the right hand (DR)						1.3944	0.3368	−1.0802	−0.9006	−0.6819
						(0.9920)	(0.9868)	(1.3196)	(1.3314)	(1.3746)
Constant	−0.9719	0.8559	2.1772	1.8303	1.9039	−1.6885*	0.6488	2.2701*	1.8987	1.6340
	(0.9481)	(0.9824)	(1.3569)	(1.3701)	(1.4109)	(0.9922)	(1.0139)	(1.3610)	(1.3752)	(1.4194)
										
Log-likelihood	−2723.0374	−2612.3722	−1429.4636	−1390.8657	−1342.9507	−2717.8522	−2608.9914	−1428.2490	−1389.6752	−1341.7123
Log-likelihood, constant term only	−2725.9650	−2725.9650	−1541.7654	−1526.7968	−1501.1664	−2723.0798	−2723.0798	−1540.3406	−1525.3701	−1499.7447
Wald chi2	5.8305	274.0802	184.4334	204.0737	246.8716	9.8131	288.6240	183.7555	202.8630	245.8082
Prob > chi2	0.1202	0.0000	0.0000	0.0000	0.0000	0.0202	0.0000	0.0000	0.0000	0.0000
Pseudo R2	0.0011	0.0417	0.0728	0.0890	0.1054	0.0019	0.0419	0.0728	0.0890	0.1054
Number of observations	2139	2139	1312	1302	1279	2137	2137	1311	1301	1278

*Note*: Robust standard errors in parentheses. Specifications (5) and (10) include secondary school regional dummies, according to settlement types. In some specifications for females STATA returns errors, outcomes with a predicted probability that is less than 0. Details available upon request.

* p<0.1,

** p<0.05,

*** p<0.01.

**Table 5:** Generalized logistic regression output, regression coefficients. Dependent variable – educational attainment. Males. The full sample.

	(1)	(2)	(3)	(4)	(5)	(6)	(7)	(8)	(9)	(10)
The first panel										
Digit ratios of the left hand (DL)	3.2277*	3.1904*	4.3532**	4.3588**	4.2502**					
	(1.6727)	(1.6685)	(2.0493)	(2.0980)	(2.1344)					
Age		−0.0159**	0.0235**	0.0256**	0.0282**		−0.0160**	0.0235**	0.0254**	0.0283**
		(0.0074)	(0.0114)	(0.0119)	(0.0132)		(0.0074)	(0.0114)	(0.0118)	(0.0132)
Education of father			1.0400***	0.6299*	0.5849			1.0490***	0.6438*	0.5987
			(0.3391)	(0.3698)	(0.3888)			(0.3386)	(0.3678)	(0.3865)
Education of mother				0.7292*	0.7144*				0.7188*	0.7098*
				(0.4021)	(0.4109)				(0.3992)	(0.4062)
										
Digit ratios of the right hand (DR)						2.0524	1.8918	2.8940	2.9893	2.7232
						(1.9608)	(1.8971)	(2.6494)	(2.7509)	(2.7463)
Constant	−1.0991	−0.3075	−3.0999	−3.1994	−3.1957	0.0649	0.9835	−1.6591	−1.8413	−1.6797
	(1.6582)	(1.7229)	(2.1621)	(2.2334)	(2.2644)	(1.9474)	(1.9338)	(2.7652)	(2.8797)	(2.8796)

The second panel										
Digit ratios of the left hand (DL)	2.6303**	2.7658**	3.6291**	3.8604**	3.3865**					
	(1.1867)	(1.1855)	(1.4907)	(1.5127)	(1.5780)					
Age		0.0074**	−0.0102	−0.0072	−0.0035		0.0070*	−0.0107	−0.0077	−0.0039
		(0.0037)	(0.0068)	(0.0069)	(0.0073)		(0.0037)	(0.0068)	(0.0069)	(0.0073)
Education of father			1.5213***	1.1709***	1.0966***			1.5256***	1.1768***	1.1037***
			(0.1805)	(0.1978)	(0.2029)			(0.1814)	(0.1988)	(0.2043)
Education of mother				0.6600***	0.6081***				0.6587***	0.6153***
				(0.2030)	(0.2104)				(0.2040)	(0.2102)
Digit ratios of the right hand (DR)						1.0218	1.0980	2.4506	2.7218*	2.4610
						(1.2609)	(1.2629)	(1.5726)	(1.6124)	(1.6646)
Constant	−2.2556*	−2.7322**	−3.2274**	−3.6096**	−3.2254**	−0.6584	−1.0590	−2.0394	−2.4616	−2.2878
	(1.1805)	(1.2020)	(1.5157)	(1.5376)	(1.6051)	(1.2561)	(1.2782)	(1.6068)	(1.6409)	(1.6927)

The third panel										
Digit ratios of the left hand (DL)	3.5982***	3.8124***	3.8739**	3.9730**	3.0852*					
	(1.2432)	(1.2432)	(1.5763)	(1.6067)	(1.6976)					
Age		0.0103***	−0.0050	−0.0005	−0.0000		0.0098***	−0.0055	−0.0011	−0.0006
		(0.0036)	(0.0071)	(0.0072)	(0.0074)		(0.0036)	(0.0071)	(0.0073)	(0.0074)
Education of father			1.6327***	1.1307***	1.0682***			1.6460***	1.1419***	1.0791***
			(0.1586)	(0.1799)	(0.1873)			(0.1588)	(0.1814)	(0.1886)
Education of mother				0.9973***	0.9882***				1.0006***	0.9920***
				(0.1885)	(0.1968)				(0.1901)	(0.1981)
Digit ratios of the right hand (DR)						2.0458	2.1745*	3.6461**	3.8292**	2.8680*
						(1.2961)	(1.2916)	(1.5745)	(1.6189)	(1.6887)
Constant	−4.0480***	−4.7395***	−4.6580***	−5.0345***	−4.1086**	−2.5031*	−3.0899**	−4.4173***	−4.8727***	−3.8707**
	(1.2399)	(1.2601)	(1.6133)	(1.6458)	(1.7440)	(1.2932)	(1.3061)	(1.6319)	(1.6710)	(1.7392)
										
Log-likelihood	−1721.6307	−1711.0707	−1085.1458	−1049.9121	−1006.2147	−1725.7357	−1715.5946	−1087.1680	−1051.9189	−1008.2145
Log-likelihood, constant term only	−1726.2623	−1726.2623	−1157.5867	−1133.1605	−1113.8247	−1727.4584	−1727.4584	−1158.6975	−1134.2828	−1114.9440
Wald chi2	9.7780	30.0863	132.1550	144.8687	4052.6961	3.1934	21.9125	129.8487	143.8310	4059.4402
Prob > chi2	0.0206	0.0000	0.0000	0.0000	0.0000	0.3628	0.0013	0.0000	0.0000	0.0000
Pseudo R2	0.0027	0.0088	0.0626	0.0735	0.0966	0.0010	0.0069	0.0617	0.0726	0.0957
Number of observations	1334	1334	912	895	880	1335	1335	913	896	881

*Note*: Robust standard errors in parentheses. Specifications (5) and (10) include secondary school regional dummies, according to settlement types.

* p<0.1,

** p<0.05,

*** p<0.01.

**Table 6:** Generalized logistic regression output, regression coefficients. Dependent variable – educational attainment. Females. The subsample created by the first method of the deletion of outliers.

	(1)	(2)	(3)	(4)	(5)	(6)	(7)	(8)	(9)	(10)
The first panel										
Digit ratios of the left hand (DL)	2.2510	−1.2185	−1.2333	−0.6585	−1.2696					
	(2.2993)	(2.2010)	(3.5893)	(3.7115)	(3.9789)					
Age		−0.0483***	0.0064	0.0103	0.0107		−0.0480***	0.0075	0.0116	0.0123
		(0.0048)	(0.0169)	(0.0172)	(0.0177)		(0.0051)	(0.0170)	(0.0176)	(0.0179)
Education of father			1.0230**	−0.0061	−0.0598			1.0206**	−0.0354	−0.1092
			(0.4407)	(0.4592)	(0.5184)			(0.4398)	(0.4647)	(0.5184)
Education of mother				2.2610***	2.9253***				2.2840***	2.9404***
				(0.7541)	(1.0502)				(0.7615)	(1.0513)
Digit ratios of the right hand (DR)						5.0461**	0.5329	1.8928	2.1946	2.2323
						(2.4015)	(2.3298)	(4.5626)	(4.7218)	(4.7674)
Constant	0.0750	6.2720***	3.9096	3.0941	3.7813	−2.7033	4.5089*	0.7361	0.1863	0.2290
	(2.2922)	(2.2761)	(3.8686)	(4.0235)	(4.2567)	(2.3866)	(2.4447)	(4.8039)	(5.0031)	(5.0270)

The second panel										
Digit ratios of the left hand (DL)	2.0257	1.7231	1.0220	1.7712	0.8834					
	(1.5207)	(1.5244)	(1.9771)	(2.0847)	(2.1185)					
Age		−0.0112***	−0.0044	0.0002	0.0060		−0.0115***	−0.0045	−0.0003	0.0062
		(0.0035)	(0.0068)	(0.0068)	(0.0073)		(0.0035)	(0.0068)	(0.0068)	(0.0073)
Education of father			1.3293***	0.6409***	0.5639**			1.3098***	0.6057***	0.5146**
			(0.1987)	(0.2246)	(0.2456)			(0.1985)	(0.2260)	(0.2457)
Education of mother				1.3271***	1.3353***				1.3248***	1.3168***
				(0.2556)	(0.2821)				(0.2578)	(0.2800)
Digit ratios of the right hand (DR)						1.5766	1.0936	−0.2666	−0.2477	−1.0547
						(1.5638)	(1.5734)	(2.1450)	(2.2195)	(2.2617)
Constant	−1.1633	−0.2884	−0.1093	−1.1461	−0.3486	−0.7142	0.3546	1.1889	0.8999	1.6032
	(1.5177)	(1.5582)	(1.9974)	(2.1202)	(2.1541)	(1.5601)	(1.6014)	(2.1632)	(2.2324)	(2.2694)

The third panel										
Digit ratios of the left hand (DL)	1.3580	0.4314	−0.8851	−1.1715	−1.2124					
	(1.4105)	(1.4426)	(1.8893)	(1.9632)	(1.9916)					
Age		−0.0243***	−0.0373***	−0.0357***	−0.0337***		−0.0241***	−0.0376***	−0.0361***	−0.0345***
		(0.0032)	(0.0061)	(0.0062)	(0.0065)		(0.0032)	(0.0061)	(0.0062)	(0.0065)
Education of father			1.5524***	1.0359***	0.9403***			1.5563***	1.0400***	0.9496***
			(0.1605)	(0.1832)	(0.1872)			(0.1603)	(0.1825)	(0.1863)
Education of mother				0.9793***	0.9480***				0.9837***	0.9603***
				(0.1925)	(0.2009)				(0.1923)	(0.2008)
Digit ratios of the right hand (DR)						2.9474**	2.0063	1.0946	1.5615	1.4145
						(1.4633)	(1.4670)	(2.0072)	(2.0632)	(2.1179)
Constant	−1.5509	0.6032	2.0273	2.1467	2.1513	−3.1365**	−0.9824	0.0582	−0.5757	−0.4621
	(1.4093)	(1.4665)	(1.9087)	(1.9899)	(2.0127)	(1.4611)	(1.4878)	(2.0256)	(2.0806)	(2.1286)
										
Log-likelihood	−2134.1649	−2053.5706	−1114.2579	−1084.7317	−1035.3825	−2131.5223	−2054.2079	−1114.5962	−1085.4547	−1035.1144
Log-likelihood, constant term only	−2135.1518	−2135.1518	−1204.7558	−1194.3461	−1173.1319	−2135.1518	−2135.1518	−1204.7558	−1194.3461	−1173.1319
Wald chi2	1.9560	206.5849	145.1253	162.2913	212.7607	7.5217	197.2079	145.8338	162.3549	213.0744
Prob > chi2	0.5816	0.0000	0.0000	0.0000	0.0000	0.0570	0.0000	0.0000	0.0000	0.0000
Pseudo R2	0.0005	0.0382	0.0751	0.0918	0.1174	0.0017	0.0379	0.0748	0.0912	0.1176
Number of observations	1710	1710	1045	1037	1019	1710	1710	1045	1037	1019

*Note*: Robust standard errors in parentheses. Specifications (5) and (10) include secondary school regional dummies, according to settlement types. In some specifications for females STATA returns errors, outcomes with a predicted probability that is less than 0. Details available upon request.

* p<0.1,

** p<0.05,

*** p<0.01.

**Table 7:** Generalized logistic regression output, regression coefficients. Dependent variable – educational attainment. Males. The subsample created by the first method of the deletion of outliers.

	(1)	(2)	(3)	(4)	(5)	(6)	(7)	(8)	(9)	(10)
The first panel										
Digit ratios of the left hand (DL)	6.6736**	6.8050**	5.9635*	5.3789	4.7567					
	(2.7734)	(2.7807)	(3.5446)	(3.5896)	(3.7691)					
Age		−0.0170*	0.0316**	0.0301**	0.0345**		−0.0164*	0.0330**	0.0311**	0.0356**
		(0.0089)	(0.0128)	(0.0135)	(0.0151)		(0.0089)	(0.0133)	(0.0140)	(0.0156)
Education of father			1.3153***	1.0765**	0.9943*			1.3484***	1.1061**	1.0325**
			(0.4509)	(0.4967)	(0.5128)			(0.4487)	(0.4897)	(0.5083)
Education of mother				0.3547	0.5321				0.3562	0.5395
				(0.4691)	(0.5065)				(0.4668)	(0.5029)
Digit ratios of the right hand (DR)						6.2213**	5.7945**	4.8418	4.8492	4.1793
						(2.6325)	(2.6037)	(3.4330)	(3.5800)	(3.7547)
Constant	−4.4701	−3.7915	−4.9594	−4.3064	−3.7800	−4.0203	−2.8179	−3.9107	−3.8287	−3.2501
	(2.7505)	(2.7481)	(3.5923)	(3.6673)	(3.7985)	(2.6122)	(2.5899)	(3.5932)	(3.7625)	(3.9287)

The second panel										
Digit ratios of the left hand (DL)	5.1183***	5.0916***	5.4015**	5.9889***	5.5467**					
	(1.7634)	(1.7615)	(2.2542)	(2.2679)	(2.3425)					
Age		0.0079*	−0.0084	−0.0058	−0.0034		0.0081*	−0.0073	−0.0046	−0.0022
		(0.0042)	(0.0077)	(0.0078)	(0.0083)		(0.0042)	(0.0077)	(0.0078)	(0.0083)
Education of father			1.4845***	1.1896***	1.1301***			1.4945***	1.2079***	1.1497***
			(0.2091)	(0.2381)	(0.2397)			(0.2097)	(0.2396)	(0.2427)
Education of mother				0.5395**	0.5056**				0.5332**	0.5046**
				(0.2428)	(0.2444)				(0.2437)	(0.2473)
Digit ratios of the right hand (DR)						4.5401**	4.5495**	5.5242**	6.3234***	6.4108**
						(1.8092)	(1.8029)	(2.3284)	(2.3962)	(2.5082)
Constant	−4.7133***	−5.0534***	−5.0171**	−5.7318**	−5.2406**	−4.1356**	−4.5199**	−5.1880**	−6.1152**	−6.1516**
	(1.7579)	(1.7663)	(2.2492)	(2.2630)	(2.3424)	(1.8025)	(1.8047)	(2.3482)	(2.4117)	(2.5228)

The third panel										
Digit ratios of the left hand (DL)	5.5133***	5.5187***	4.7503**	5.2349**	4.2362*					
	(1.7785)	(1.7807)	(2.1809)	(2.1948)	(2.2970)					
Age		0.0094**	−0.0043	0.0001	−0.0016		0.0095**	−0.0043	0.0001	−0.0016
		(0.0041)	(0.0081)	(0.0082)	(0.0085)		(0.0041)	(0.0081)	(0.0083)	(0.0086)
Education of father			1.5467***	1.0664***	1.0433***			1.5644***	1.0832***	1.0662***
			(0.1820)	(0.2126)	(0.2256)			(0.1824)	(0.2167)	(0.2307)
Education of mother				0.8986***	0.8746***				0.9031***	0.8754***
				(0.2232)	(0.2337)				(0.2266)	(0.2391)
Digit ratios of the right hand (DR)						5.3085***	5.3024***	7.3764***	7.9591***	7.6120***
						(1.8632)	(1.8608)	(2.3121)	(2.3437)	(2.5496)
Constant	−5.9758***	−6.4221***	−5.5279**	−6.2702***	−5.0727**	−5.7690***	−6.2048***	−8.1563***	−8.9959***	−8.4450***
	(1.7772)	(1.7890)	(2.1850)	(2.1990)	(2.3110)	(1.8603)	(1.8646)	(2.3457)	(2.3688)	(2.5766)
										
Log-likelihood	−1298.7501	−1290.7834	−816.0790	−791.5661	−750.9552	−1299.7907	−1292.0505	−814.7216	−789.9952	−749.0929
Log-likelihood, constant term only	−1304.7007	−1304.7007	−868.7191	−849.8762	−833.7735	−1304.7007	−1304.7007	−868.7191	−849.8762	−833.7735
Wald chi2	12.9592	26.8157	95.1559	101.9754	3470.2519	11.1928	24.6464	99.1496	107.5438	3035.8692
Prob > chi2	0.0047	0.0002	0.0000	0.0000	0.0000	0.0107	0.0004	0.0000	0.0000	0.0000
Pseudo R2	0.0046	0.0107	0.0606	0.0686	0.0993	0.0038	0.0097	0.0622	0.0705	0.1016
Number of observations	1010	1010	688	675	663	1010	1010	688	675	663

*Note*: Robust standard errors in parentheses. Specifications (5) and (10) include secondary school regional dummies, according to settlement types.

* p<0.1,

** p<0.05,

*** p<0.01.

**Table 8:** Generalized logistic regression output, regression coefficients. Dependent variable – educational attainment. Females. The subsample created by the second method of the deletion of outliers.

	(1)	(2)	(3)	(4)	(5)	(6)	(7)	(8)	(9)	(10)
The first panel										
Digit ratios of the left hand (DL)	3.6392**	0.5897	0.8193	0.6356	0.6645					
	(1.5274)	(1.5654)	(2.4705)	(2.5275)	(2.5905)					
Age		−0.0491***	0.0056	0.0083	0.0106		−0.0491***	0.0053	0.0079	0.0103
		(0.0041)	(0.0139)	(0.0145)	(0.0144)		(0.0041)	(0.0141)	(0.0147)	(0.0146)
Education of father			0.9800***	0.0925	0.0044			0.9750**	0.0841	−0.0055
			(0.3799)	(0.4303)	(0.4702)			(0.3812)	(0.4298)	(0.4694)
Education of mother				1.9575***	2.3490***				1.9582***	2.3445***
				(0.6733)	(0.8387)				(0.6714)	(0.8370)
Digit ratios of the right hand (DR)						2.8032*	0.2486	−0.4377	−0.8620	−0.6707
						(1.6125)	(1.3376)	(2.8849)	(2.9935)	(2.9975)
Constant	−1.4175	4.4127***	1.8291	1.8360	1.8413	−0.5896	4.7529***	3.0990	3.3559	3.1938
	(1.5163)	(1.6359)	(2.7317)	(2.8057)	(2.8506)	(1.6040)	(1.4018)	(3.1556)	(3.2706)	(3.2526)

The second panel										
Digit ratios of the left hand (DL)	1.5186	1.1237	−0.2535	−0.0692	−0.3430					
	(1.0527)	(1.0557)	(1.3516)	(1.3855)	(1.4235)					
Age		−0.0127***	−0.0059	−0.0014	0.0015		−0.0132***	−0.0060	−0.0016	0.0014
		(0.0030)	(0.0059)	(0.0059)	(0.0062)		(0.0030)	(0.0059)	(0.0059)	(0.0062)
Education of father			1.4004***	0.7479***	0.6993***			1.3989***	0.7388***	0.6902***
			(0.1757)	(0.2157)	(0.2283)			(0.1759)	(0.2145)	(0.2267)
Education of mother				1.2681***	1.2489***				1.2749***	1.2518***
				(0.2453)	(0.2630)				(0.2449)	(0.2627)
Digit ratios of the right hand (DR)						0.3955	−0.2107	−0.9362	−0.8900	−1.0166
						(1.0554)	(1.0290)	(1.3306)	(1.3702)	(1.3885)
Constant	−0.7482	0.3009	1.1315	0.6727	0.9111	0.3720	1.6571	1.8230	1.5030	1.5948
	(1.0504)	(1.0801)	(1.3887)	(1.4248)	(1.4633)	(1.0549)	(1.0620)	(1.3749)	(1.4132)	(1.4293)

The third panel										
Digit ratios of the left hand (DL)	1.1969	0.6018	−0.8619	−0.7802	−0.8674					
	(0.9942)	(1.0178)	(1.3375)	(1.3526)	(1.3922)					
Age		−0.0250***	−0.0402***	−0.0381***	−0.0367***		−0.0251***	−0.0403***	−0.0383***	−0.0368***
		(0.0028)	(0.0055)	(0.0055)	(0.0057)		(0.0029)	(0.0055)	(0.0055)	(0.0057)
Education of father			1.5018***	0.9980***	0.9359***			1.5067***	1.0030***	0.9406***
			(0.1398)	(0.1612)	(0.1630)			(0.1400)	(0.1612)	(0.1628)
Education of mother				0.9928***	0.9516***				0.9930***	0.9517***
				(0.1721)	(0.1764)				(0.1718)	(0.1760)
Digit ratios of the right hand (DR)						1.6498*	0.5852	−0.9736	−0.7972	−0.5114
						(1.0014)	(1.0009)	(1.3258)	(1.3395)	(1.3756)
Constant	−1.4855	0.3725	2.0334	1.7626	1.8020	−1.9389*	0.3890	2.1517	1.7843	1.4482
	(0.9936)	(1.0380)	(1.3731)	(1.3919)	(1.4295)	(1.0015)	(1.0274)	(1.3667)	(1.3826)	(1.4192)
										
Log−likelihood	−2698.4541	−2590.8460	−1420.2816	−1382.6169	−1333.9520	−2698.0187	−2590.9686	−1420.2873	−1382.6404	−1333.9385
Log-likelihood, constant term only	−2701.5398	−2701.5398	−1533.1370	−1518.1611	−1492.5486	−2701.5398	−2701.5398	−1533.1370	−1518.1611	−1492.5486
Wald chi2	5.9076	267.0128	184.5987	202.7915	246.9398	6.7064	269.0797	184.4922	201.9166	246.1988
Prob > chi2	0.1162	0.0000	0.0000	0.0000	0.0000	0.0819	0.0000	0.0000	0.0000	0.0000
Pseudo R2	0.0011	0.0410	0.0736	0.0893	0.1063	0.0013	0.0409	0.0736	0.0893	0.1063
Number of observations	2122	2122	1305	1295	1272	2122	2122	1305	1295	1272

*Note*: Robust standard errors in parentheses. Specifications (5) and (10) include secondary school regional dummies, according to settlement types. In some specifications for females STATA returns errors, outcomes with a predicted probability that is less than 0. Details available upon request.

* p<0.1,

** p<0.05,

*** p<0.01.

**Table 9:** Generalized logistic regression output, regression coefficients. Dependent variable – educational attainment. Males. The subsample created by the second method of the deletion of outliers.

	(1)	(2)	(3)	(4)	(5)	(6)	(7)	(8)	(9)	(10)
The first panel										
Digit ratios of the left hand (DL)	3.7177**	3.7060**	4.5882**	4.5931**	4.5483*					
	(1.8033)	(1.7935)	(2.2423)	(2.2980)	(2.3401)					
Age		−0.0153**	0.0233**	0.0254**	0.0284**		−0.0154**	0.0234**	0.0253**	0.0284**
		(0.0074)	(0.0114)	(0.0118)	(0.0133)		(0.0075)	(0.0114)	(0.0119)	(0.0133)
Education of father			1.0407***	0.6251*	0.5737			1.0478***	0.6369*	0.5879
			(0.3392)	(0.3696)	(0.3875)			(0.3389)	(0.3672)	(0.3843)
Education of mother				0.7401*	0.7389*				0.7304*	0.7372*
				(0.4018)	(0.4126)				(0.3984)	(0.4072)
Digit ratios of the right hand (DR)						2.4277	2.2814	2.9278	3.0479	2.6889
						(1.9671)	(1.9028)	(2.6664)	(2.7638)	(2.7851)
Constant	−1.5817	−0.8454	−3.3298	−3.4310	−3.5043	−0.3058	0.5664	−1.6932	−1.9014	−1.6563
	(1.7861)	(1.8372)	(2.3277)	(2.4055)	(2.4461)	(1.9527)	(1.9332)	(2.7753)	(2.8849)	(2.9055)

The second panel										
Digit ratios of the left hand (DL)	2.6709**	2.7917**	3.6392**	3.8688**	3.5457**					
	(1.2247)	(1.2238)	(1.5565)	(1.5803)	(1.6353)					
Age		0.0073**	−0.0105	−0.0076	−0.0042		0.0071*	−0.0106	−0.0076	−0.0041
		(0.0037)	(0.0068)	(0.0069)	(0.0073)		(0.0037)	(0.0068)	(0.0069)	(0.0073)
Education of father			1.5127***	1.1586***	1.0845***			1.5104***	1.1565***	1.0841***
			(0.1806)	(0.1979)	(0.2035)			(0.1814)	(0.1988)	(0.2048)
Education of mother				0.6647***	0.6085***				0.6667***	0.6191***
				(0.2029)	(0.2109)				(0.2039)	(0.2108)
Digit ratios of the right hand (DR)						1.3710	1.4340	2.7588*	3.0880*	2.8176*
						(1.2634)	(1.2650)	(1.5855)	(1.6102)	(1.6697)
Constant	−2.2943*	−2.7529**	−3.2225**	−3.6019**	−3.3585**	−1.0025	−1.3933	−2.3444	−2.8256*	−2.6326
	(1.2185)	(1.2377)	(1.5753)	(1.5987)	(1.6580)	(1.2584)	(1.2785)	(1.6179)	(1.6375)	(1.6970)

The third panel										
Digit ratios of the left hand (DL)	3.6609***	3.8546***	3.9814**	4.0698**	3.2953*					
	(1.2639)	(1.2636)	(1.6065)	(1.6375)	(1.7269)					
Age		0.0103***	−0.0055	−0.0011	−0.0009		0.0100***	−0.0058	−0.0015	−0.0012
		(0.0036)	(0.0071)	(0.0072)	(0.0074)		(0.0036)	(0.0071)	(0.0073)	(0.0074)
Education of father			1.6196***	1.1126***	1.0485***			1.6291***	1.1171***	1.0546***
			(0.1589)	(0.1803)	(0.1882)			(0.1589)	(0.1813)	(0.1891)
Education of mother				1.0026***	0.9923***				1.0137***	1.0026***
				(0.1886)	(0.1975)				(0.1901)	(0.1988)
Digit ratios of the right hand (DR)						2.5251*	2.6368**	4.2585***	4.5633***	3.6360**
						(1.2941)	(1.2883)	(1.6025)	(1.5953)	(1.6993)
Constant	−4.1062***	−4.7798***	−4.7393***	−5.1039***	−4.2833**	−2.9756**	−3.5534***	−5.0085***	−5.5864***	−4.6097***
	(1.2605)	(1.2787)	(1.6398)	(1.6729)	(1.7703)	(1.2913)	(1.3015)	(1.6583)	(1.6492)	(1.7500)
										
Log-likelihood	−1712.6158	−1702.4898	−1081.3821	−1046.0376	−1001.9992	−1715.0958	−1705.2487	−1081.9166	−1046.3163	−1002.6716
Log-likelihood, constant term only	−1717.5064	−1717.5064	−1152.8126	−1128.3866	−1109.0594	−1717.5064	−1717.5064	−1152.8126	−1128.3866	−1109.0594
Wald chi2	10.1074	29.5783	129.9453	142.9787	4028.5530	4.6051	22.8611	128.6178	143.0427	4052.1626
Prob > chi2	0.0177	0.0000	0.0000	0.0000	0.0000	0.2031	0.0008	0.0000	0.0000	0.0000
Pseudo R2	0.0028	0.0087	0.0620	0.0730	0.0965	0.0014	0.0071	0.0615	0.0727	0.0959
Number of observations	1328	1328	908	891	876	1328	1328	908	891	876

*Note*: Robust standard errors in parentheses. Specifications (5) and (10) include secondary school regional dummies, according to settlement types.

* p<0.1,

** p<0.05,

*** p<0.01.

**Table 10:** Self-identified nationalities.

Self-identified nationality	Groups for dummy variables	Freq.	Percent
**Russian**	**Reference**	3174	90.02
Ukrainian	Reference	44	1.25
Belarusian	Reference	18	0.51
German	Reference	2	0.06
Polish	Reference	4	0.11
Armenian	Caucasian	47	1.33
Tatar	Reference	61	1.73
Spain	Reference	1	0.03
Chuvash	Reference	14	0.4
Moldavian	Reference	14	0.4
Udmurt	Reference	2	0.06
Mordvinian	Reference	14	0.4
Azerbaijan	Caucasian	14	0.4
Kazakh	Asian	2	0.06
Jewish	Reference	27	0.77
Bashkir	Reference	1	0.03
Lezghian	Caucasian	4	0.11
Lithuanian	Reference	4	0.11
Kabardinian	Caucasian	1	0.03
Dargin	Caucasian	1	0.03
Ossetian	Caucasian	1	0.03
Georgian	Caucasian	13	0.37
Turkmen	Asian	1	0.03
Korean	Asian	5	0.14
Bulgarian	Reference	3	0.09
Uzbek	Asian	17	0.48
Avar	Caucasian	5	0.14
Karelian	Reference	1	0.03
Kirghiz	Asian	2	0.06
Afghean	Reference	1	0.03
Ingush	Caucasian	5	0.14
Daghestani	Caucasian	1	0.03
Persian	Reference	1	0.03
Tajik	Asian	9	0.26
Turkish	Reference	1	0.03
Chechen	Caucasian	1	0.03
Serbian	Reference	1	0.03
Cossack	Reference	1	0.03
Indian	Reference	1	0.03
Kalmyk	Reference	3	0.09
Yazidi	Reference	3	0.09
Circassian	Caucasian	1	0.03
Total		3526	100

*Note*: The original RLMS question for the self-identification of individuals’ nationality is the following: “What nationality do you consider yourself? I don’t necessarily have in mind the nationality that is indicated on your passport.” Please, consult: http://www.cpc.unc.edu/projects/rlms-hse/data/questionnaires/rtadult.pdf

**Table 11:** Generalized logistic regression output, regression coefficients. Dependent variable – educational attainment. Males. The sample of Tatars, Armenians and Ukrainians.

	(1)	(2)	(3)	(4)	(5)	(6)	(7)	(8)	(9)	(10)
1										
Digit ratios of the left hand (DL)	−3.2129	−3.3838	1.4044	7.2388	138.2858***					
	(4.4018)	(5.0976)	(6.0398)	(7.7933)	(33.0610)					
Age		−0.0159	−0.0137	−0.0104	2.8811***		−0.0337	−0.0103	0.0004	3.7826***
		(0.0286)	(0.0331)	(0.0393)	(0.2693)		(0.0315)	(0.0675)	(0.0748)	(0.2636)
Education of father			14.6014***	17.7233***	27.8663***			14.0499***	14.6404***	29.9446***
			(0.9194)	(1.1132)	(2.9015)			(1.5259)	(1.8320)	(3.4283)
Education of mother				−3.31e+08	−45.2892***				−1.23e+05	−68.8324***
				(.)	(5.3531)				(.)	(2.9164)
Digit ratios of the right hand (DR)						−12.4537	−16.7074	−11.5404	−9.7312	165.3568***
						(9.5852)	(13.4188)	(19.0085)	(21.9590)	(40.7698)
Constant	4.7156	5.7065	0.8530	−5.0125	−179.9617***	14.0107	19.9862	13.8269	11.6191	−230.9693***
	(4.4133)	(6.3320)	(6.1458)	(7.4712)	(29.3040)	(9.7727)	(14.8632)	(21.2625)	(24.1982)	(43.6102)
										
2										
Digit ratios of the left hand (DL)	−0.5718	−0.2433	−0.8594	0.7721	−7.5969					
	(4.8433)	(4.7001)	(8.1895)	(9.4463)	(12.5775)					
Age		−0.0119	−0.0653	−0.0447	−0.0020		−0.0112	−0.0901*	−0.0617	−0.0092
		(0.0194)	(0.0442)	(0.0458)	(0.1461)		(0.0201)	(0.0470)	(0.0484)	(0.1016)
Education of father			1.9362*	1.1651	18.2165***			2.8778***	2.4399**	20.5987***
			(1.0460)	(1.1304)	(1.9342)			(0.9603)	(1.0494)	(1.5087)
Education of mother				16.6617***	40.4975***				16.5196***	43.1153***
				(1.3449)	(2.5600)				(0.9055)	(2.2779)
Digit ratios of the right hand (DR)						13.0330**	14.2040**	16.4497**	17.4375**	21.2544
						(5.5658)	(5.7483)	(8.3079)	(8.7228)	(21.7786)
Constant	0.6773	0.9548	3.5236	1.0055	8.0650	−12.9992**	−13.5861**	−13.1105	−15.3013	−19.5196
	(4.8637)	(4.8888)	(8.3808)	(9.5518)	(15.3778)	(5.6532)	(5.8584)	(8.8615)	(9.4117)	(22.3765)
										
3										
Digit ratios of the left hand (DL)	14.8990*	14.1590	32.7083**	56.7455**	71.2787**					
	(8.0110)	(9.7392)	(15.5682)	(23.2198)	(34.5274)					
Age		−0.0054	0.0004	−0.0260	−0.0199		0.0133	0.0166	0.0152	0.0979
		(0.0247)	(0.0634)	(0.0756)	(0.1809)		(0.0224)	(0.0524)	(0.0622)	(0.1794)
Education of father			0.5109	−1.1655	−37.2333***			1.1969	0.4871	−38.0722***
			(0.8679)	(0.7749)	(1.6169)			(0.8007)	(0.8347)	(1.6102)
Education of mother				4.5829***	57.8446***				2.0891*	60.6449***
				(1.6984)	(1.8183)				(1.1006)	(3.5608)
Digit ratios of the right hand (DR)						13.1946*	12.9545	14.3050	14.8098	16.4022
						(7.4544)	(8.1298)	(11.9358)	(12.6759)	(11.0959)
Constant	−15.5535*	−14.5482	−33.4480**	−56.8495**	−69.8195*	−13.9784*	−14.3420*	−15.9229	−16.5215	−19.8110
	(8.1603)	(9.2587)	(15.5690)	(24.5713)	(37.7463)	(7.5476)	(7.8269)	(11.7857)	(12.9564)	(13.9092)
										
Log-likelihood	−71.5281	−71.2048	−35.0046	−27.7305	−12.6871	−69.2130	−67.6023	−35.1905	−30.6514	−13.4989
Log-likelihood, constant term only	−75.0919	−75.0919	−43.0988	−41.7571	−39.5565	−75.0919	−75.0919	−43.0988	−41.7571	−39.5565
Wald chi2	4.7266	5.3011	527.0742	.	.	10.0497	11.8920	461.3394	.	.
Prob > chi2	0.1929	0.5058	0.0000	.	.	0.0181	0.0644	0.0000	.	.
Pseudo R2	0.0475	0.0518	0.1878	0.3359	0.6793	0.0783	0.0997	0.1835	0.2660	0.6587
Number of observations	56	56	33	32	31	56	56	33	32	31

*Note*: Robust standard errors. Specifications (5) and (10) include secondary school regional dummies, according to settlement types.

* p<0.1,

** p<0.05,

*** p<0.01.

**Table 12:** Generalized logistic regression output, regression coefficients. Self-identities of nationalities are included. Dependent variable – educational attainment. Females. The full sample.

	(1)	(2)	(3)	(4)	(5)	(6)	(7)	(8)	(9)	(10)
The first panel										
Digit ratios of the left hand (DL)	3.4694**	0.5777	0.7577	0.6873	1.0052					
	(1.4557)	(1.4411)	(2.3646)	(2.3772)	(2.3964)					
Asian	0.0511	−1.0031	−0.8880	−1.0064	−1.1457	0.1344	−1.0038	−0.9139	−1.0601	−1.1831
	(0.7439)	(0.7849)	(0.7701)	(0.7702)	(0.8540)	(0.7451)	(0.7841)	(0.7851)	(0.7874)	(0.8664)
Caucasian	0.5336	−0.0870	−0.6753	−0.6317	−0.7905	0.5657	−0.0833	−0.6892	−0.6500	−0.7749
	(0.5974)	(0.6198)	(0.6281)	(0.6225)	(0.6586)	(0.6036)	(0.6207)	(0.6317)	(0.6256)	(0.6589)
Age		−0.0502***	0.0032	0.0056	0.0068		−0.0500***	0.0028	0.0049	0.0062
		(0.0041)	(0.0140)	(0.0146)	(0.0146)		(0.0041)	(0.0142)	(0.0148)	(0.0150)
Education of father			0.9626**	0.1159	0.0514			0.9658**	0.1175	0.0716
			(0.3823)	(0.4302)	(0.4681)			(0.3837)	(0.4282)	(0.4661)
Education of mother				1.9050***	2.3045***				1.8970***	2.2803***
				(0.6641)	(0.8234)				(0.6612)	(0.8208)
Digit ratios of the right hand (DR)						3.2302**	0.4036	−0.9750	−1.4732	−1.3620
						(1.5104)	(1.2777)	(2.7860)	(2.8479)	(2.8443)
Constant	−1.2696	4.4928***	2.0362	1.9481	1.6599	−1.0344	4.6532***	3.7906	4.1479	4.0564
	(1.4451)	(1.5066)	(2.6020)	(2.6344)	(2.6184)	(1.5010)	(1.3453)	(3.0610)	(3.1207)	(3.0988)

The second panel										
Digit ratios of the left hand (DL)	1.0186	0.6306	−0.5326	−0.3329	−0.6445					
	(0.9967)	(0.9967)	(1.3589)	(1.3770)	(1.4116)					
Asian	−0.2435	−0.4052	−0.3657	−0.3697	−0.1175	−0.2645	−0.4415	−0.4091	−0.4202	−0.1678
	(0.4559)	(0.4761)	(0.4796)	(0.4964)	(0.5283)	(0.4524)	(0.4728)	(0.4861)	(0.4993)	(0.5318)
Caucasian	0.1413	0.0838	−0.2887	−0.2819	−0.4025	0.1379	0.0689	−0.3224	−0.3137	−0.4356
	(0.3208)	(0.3329)	(0.3812)	(0.3781)	(0.3895)	(0.3216)	(0.3343)	(0.3837)	(0.3799)	(0.3910)
Age		−0.0132***	−0.0074	−0.0028	0.0006		−0.0138***	−0.0076	−0.0031	0.0004
		(0.0030)	(0.0058)	(0.0059)	(0.0061)		(0.0030)	(0.0058)	(0.0059)	(0.0062)
Education of father			1.3534***	0.7066***	0.6400***			1.3485***	0.6953***	0.6290***
			(0.1728)	(0.2095)	(0.2215)			(0.1730)	(0.2085)	(0.2204)
Education of mother				1.2781***	1.2879***				1.2829***	1.2907***
				(0.2429)	(0.2604)				(0.2423)	(0.2600)
Digit ratios of the right hand (DR)						−0.1016	−0.7059	−1.3531	−1.2988	−1.4099
						(1.0424)	(1.0033)	(1.3517)	(1.3855)	(1.4040)
Constant	−0.2737	0.7935	1.4724	0.9945	1.2498	0.8460	2.1624**	2.3113*	1.9791	2.0331
	(0.9956)	(1.0213)	(1.3987)	(1.4175)	(1.4519)	(1.0432)	(1.0382)	(1.4014)	(1.4329)	(1.4473)

The third panel										
Digit ratios of the left hand (DL)	0.6088	0.0551	−1.0421	−0.9271	−1.0615					
	(0.9531)	(0.9669)	(1.3278)	(1.3416)	(1.3759)					
Asian	−0.3807	−0.5706	−0.3968	−0.3151	−0.2452	−0.3420	−0.5626	−0.4216	−0.3346	−0.2524
	(0.4659)	(0.4661)	(0.4835)	(0.5110)	(0.5241)	(0.4647)	(0.4663)	(0.4874)	(0.5139)	(0.5243)
Caucasian	0.0541	−0.0119	−0.2958	−0.3225	−0.6696*	0.0716	−0.0078	−0.3203	−0.3424	−0.6853*
	(0.2947)	(0.3055)	(0.3696)	(0.3734)	(0.3914)	(0.2944)	(0.3057)	(0.3703)	(0.3738)	(0.3915)
Age		−0.0254***	−0.0412***	−0.0392***	−0.0378***		−0.0255***	−0.0415***	−0.0394***	−0.0380***
		(0.0029)	(0.0055)	(0.0055)	(0.0057)		(0.0029)	(0.0055)	(0.0055)	(0.0057)
Education of father			1.4724***	0.9874***	0.9110***			1.4754***	0.9912***	0.9134***
			(0.1396)	(0.1608)	(0.1625)			(0.1398)	(0.1608)	(0.1623)
Education of mother				0.9711***	0.9607***				0.9698***	0.9594***
				(0.1730)	(0.1767)				(0.1727)	(0.1763)
Digit ratios of the right hand (DR)						1.3266	0.2235	−1.3007	−1.0926	−0.9749
						(0.9996)	(0.9963)	(1.3366)	(1.3530)	(1.3905)
Constant	−0.9143	0.9262	2.2596*	1.9595	2.0386	−1.6308	0.7600	2.5314*	2.1359	1.9614
	(0.9531)	(0.9880)	(1.3651)	(1.3819)	(1.4142)	(1.0004)	(1.0247)	(1.3822)	(1.4004)	(1.4368)
										
Log-likelihood	−2706.9546	−2596.6330	−1418.8146	−1381.4348	−1336.1671	−2701.6436	−2593.2432	−1417.4354	−1380.0944	−1334.8842
Log-likelihood, constant term only	−2710.7288	−2710.7288	−1531.4485	−1516.5009	−1496.0112	−2707.8530	−2707.8530	−1530.0329	−1515.0835	−1494.5942
Wald chi2	7.6352	280.9926	186.4528	204.7638	251.2461	11.5813	296.2479	185.8479	203.5297	250.6411
Prob > chi2	0.5713	0.0000	0.0000	0.0000	0.0000	0.2380	0.0000	0.0000	0.0000	0.0000
Pseudo R2	0.0014	0.0421	0.0735	0.0891	0.1068	0.0023	0.0423	0.0736	0.0891	0.1069
Number of observations	2123	2123	1300	1290	1273	2121	2121	1299	1289	1272

*Note*: Robust standard errors in parentheses. Specifications (5) and (10) include secondary school regional dummies, according to settlement types. Individuals which are not belong to “Asian” or “Caucasian” groups constitute the reference group. In some specifications for females STATA returns errors, outcomes with a predicted probability that is less than 0. Details available upon request.

* p<0.1,

** p<0.05,

*** p<0.01.

**Table 13:** Generalized logistic regression output, regression coefficients. Self-identities of nationalities are included. Dependent variable – educational attainment. Males. The full sample.

	(1)	(2)	(3)	(4)	(5)	(6)	(7)	(8)	(9)	(10)
The first panel										
Digit ratios of the left hand (DL)	3.4420**	3.3745**	4.5063**	4.4978**	4.4869**					
	(1.6427)	(1.6546)	(1.9980)	(2.0265)	(2.0938)					
Asian	14.4238***	12.6591***	13.1683***	13.4548***	14.7539***	14.3990***	12.6323***	12.5111***	13.1712***	14.7322***
	(0.2758)	(0.3112)	(0.3554)	(0.3936)	(0.5079)	(0.2765)	(0.3122)	(0.3510)	(0.3883)	(0.5056)
Caucasian	−0.3322	−0.3377	−0.7512	−0.5965	−0.7328	−0.3679	−0.3698	−0.8138*	−0.6744	−0.8112
	(0.4533)	(0.4571)	(0.4914)	(0.5553)	(0.5817)	(0.4521)	(0.4546)	(0.4941)	(0.5598)	(0.5754)
Age		−0.0159**	0.0262**	0.0273**	0.0310**		−0.0160**	0.0263**	0.0273**	0.0311**
		(0.0075)	(0.0117)	(0.0121)	(0.0137)		(0.0076)	(0.0117)	(0.0121)	(0.0136)
Education of father			1.0037***	0.6222*	0.5963			1.0110***	0.6375*	0.6122
			(0.3413)	(0.3782)	(0.4004)			(0.3408)	(0.3769)	(0.3985)
Education of mother				0.6579	0.6217				0.6412	0.6096
				(0.4090)	(0.4248)				(0.4059)	(0.4208)
Digit ratios of the right hand (DR)						2.2859	2.0980	3.2457	3.2677	3.1124
						(1.9378)	(1.8889)	(2.6154)	(2.6946)	(2.6873)
Constant	−1.3132	−0.4899	−3.3441	−3.3981	−3.5216	−0.1675	0.7795	−2.1011	−2.1783	−2.1555
	(1.6291)	(1.7183)	(2.1223)	(2.1735)	(2.2390)	(1.9244)	(1.9348)	(2.7408)	(2.8302)	(2.8236)

The second panel										
Digit ratios of the left hand (DL)	2.7348**	2.8747**	3.5873**	3.8184**	3.4240**					
	(1.1906)	(1.1902)	(1.4936)	(1.5197)	(1.5824)					
Asian	0.0837	0.1703	0.0396	−0.1412	0.1483	0.0548	0.1351	0.0233	−0.1635	0.1389
	(0.5254)	(0.5258)	(0.6213)	(0.6540)	(0.7804)	(0.5296)	(0.5312)	(0.6386)	(0.6724)	(0.8054)
Caucasian	0.2035	0.1962	0.0479	0.2754	−0.0334	0.1870	0.1796	−0.0002	0.2119	−0.1124
	(0.3301)	(0.3321)	(0.3467)	(0.3813)	(0.4224)	(0.3294)	(0.3312)	(0.3390)	(0.3725)	(0.4165)
Age		0.0071*	−0.0106	−0.0079	−0.0036		0.0067*	−0.0111	−0.0084	−0.0039
		(0.0037)	(0.0069)	(0.0070)	(0.0074)		(0.0037)	(0.0069)	(0.0070)	(0.0074)
Education of father			1.5093***	1.1425***	1.0721***			1.5158***	1.1515***	1.0835***
			(0.1815)	(0.1996)	(0.2051)			(0.1823)	(0.2007)	(0.2063)
Education of mother				0.6801***	0.6156***				0.6765***	0.6209***
				(0.2029)	(0.2122)				(0.2039)	(0.2125)
Digit ratios of the right hand (DR)						1.0349	1.1143	2.4039	2.5891	2.5273
						(1.2725)	(1.2744)	(1.5919)	(1.6317)	(1.6753)
Constant	−2.3700**	−2.8404**	−3.1766**	−3.5556**	−3.2519**	−0.6812	−1.0733	−1.9846	−2.3165	−2.3453
	(1.1851)	(1.2084)	(1.5203)	(1.5463)	(1.6097)	(1.2679)	(1.2915)	(1.6312)	(1.6635)	(1.7016)

The third panel										
Digit ratios of the left hand (DL)	3.5364***	3.7668***	3.6661**	3.7891**	2.9513*					
	(1.2471)	(1.2483)	(1.5865)	(1.6203)	(1.7081)					
Asian	0.0960	0.2314	−0.0006	−0.1059	0.0988	0.1000	0.2302	0.0419	−0.0672	0.1168
	(0.5176)	(0.5090)	(0.6638)	(0.5734)	(0.6347)	(0.5153)	(0.5087)	(0.6749)	(0.5838)	(0.6514)
Caucasian	−0.0848	−0.0848	−0.1802	−0.0184	−0.6796	−0.1101	−0.1113	−0.2331	−0.0791	−0.7392
	(0.3223)	(0.3188)	(0.4330)	(0.4567)	(0.5212)	(0.3285)	(0.3253)	(0.4327)	(0.4580)	(0.5247)
Age		0.0105***	−0.0048	−0.0005	0.0006		0.0101***	−0.0051	−0.0010	0.0001
		(0.0036)	(0.0071)	(0.0073)	(0.0075)		(0.0036)	(0.0072)	(0.0074)	(0.0075)
Education of father			1.6614***	1.1454***	1.1100***			1.6769***	1.1587***	1.1250***
			(0.1597)	(0.1816)	(0.1891)			(0.1602)	(0.1834)	(0.1911)
Education of mother				1.0143***	0.9649***				1.0163***	0.9668***
				(0.1894)	(0.1983)				(0.1909)	(0.1998)
Digit ratios of the right hand (DR)						2.0531	2.1976*	3.6478**	3.7838**	3.0431*
						(1.3071)	(1.3035)	(1.6058)	(1.6538)	(1.7021)
Constant	−3.9884***	−4.7101***	−4.4765***	−4.8746***	−4.0018**	−2.5118*	−3.1284**	−4.4502***	−4.8548***	−4.0746**
	(1.2445)	(1.2670)	(1.6255)	(1.6614)	(1.7538)	(1.3045)	(1.3195)	(1.6717)	(1.7128)	(1.7603)
										
Log-likelihood	−1706.0219	−1695.6492	−1073.8227	−1038.6709	−998.9212	−1710.0307	−1700.0752	−1075.6429	−1040.5814	−1000.7553
Log-likelihood, constant term only	−1713.7000	−1713.7000	−1150.0360	−1125.6622	−1110.1811	−1714.8936	−1714.8936	−1151.1423	−1126.7802	−1111.3004
Wald chi2	2774.2984	2171.1505	1604.0328	1615.9228	5262.5675	2765.0489	2150.4716	1498.7610	1682.0764	5319.5344
Prob > chi2	0.0000	0.0000	0.0000	0.0000	0.0000	0.0000	0.0000	0.0000	0.0000	0.0000
Pseudo R2	0.0045	0.0105	0.0663	0.0773	0.1002	0.0028	0.0086	0.0656	0.0765	0.0995
Number of observations	1324	1324	905	888	877	1325	1325	906	889	878

*Note*: Robust standard errors in parentheses. Specifications (5) and (10) include secondary school regional dummies, according to settlement types. Individuals which are not belong to “Asian” or “Caucasian” groups constitute the reference group.

* p<0.1,

** p<0.05,

*** p<0.01.

**Table 14:** Generalized logistic regression output, regression coefficients. Self-identities of nationalities are included. Dependent variable – educational attainment. Females. The subsample created by the first method of the deletion of outliers.

	(1)	(2)	(3)	(4)	(5)	(6)	(7)	(8)	(9)	(10)
The first panel										
Digit ratios of the left hand (DL)	2.3054	−1.2371	−1.2778	−0.7108	−1.3931					
	(2.3051)	(2.1966)	(3.5502)	(3.6452)	(3.9067)					
Asian	0.4305	−0.5139	−0.3691	−0.5659	−0.5436	0.4565	−0.5175	−0.3114	−0.5186	−0.4556
	(1.0320)	(1.0766)	(1.0486)	(1.0485)	(1.1853)	(1.0479)	(1.0788)	(1.0718)	(1.0772)	(1.2127)
Caucasian	1.2099	0.6945	0.0801	0.0181	−0.1433	1.3018	0.7160	0.1190	0.0825	−0.0609
	(1.0171)	(1.0254)	(1.0361)	(1.0381)	(1.0648)	(1.0190)	(1.0262)	(1.0438)	(1.0473)	(1.0676)
Age		−0.0484***	0.0055	0.0090	0.0092		−0.0480***	0.0068	0.0105	0.0111
		(0.0048)	(0.0169)	(0.0172)	(0.0180)		(0.0051)	(0.0172)	(0.0177)	(0.0182)
Education of father			1.0062**	−0.0209	−0.0881			1.0055**	−0.0549	−0.1230
			(0.4458)	(0.4595)	(0.5175)			(0.4424)	(0.4644)	(0.5166)
Education of mother				2.2566***	2.8898***				2.2863***	2.9243***
				(0.7534)	(1.0463)				(0.7598)	(1.0480)
Digit ratios of the right hand (DR)						5.2527**	0.5250	1.7431	1.9417	2.0526
						(2.4108)	(2.3479)	(4.6745)	(4.8489)	(4.8488)
Constant	−0.0043	6.2824***	3.9925	3.2084	3.9753	−2.9353	4.5068*	0.9144	0.4922	0.4632
	(2.2974)	(2.2678)	(3.8179)	(3.9450)	(4.1794)	(2.3968)	(2.4672)	(4.9488)	(5.1599)	(5.1457)

The second panel										
Digit ratios of the left hand (DL)	2.0047	1.6750	1.0075	1.7202	0.8490					
	(1.5214)	(1.5245)	(1.9810)	(2.0907)	(2.1084)					
Asian	−0.3188	−0.4307	−0.2399	−0.3073	0.1721	−0.3156	−0.4312	−0.2651	−0.3621	0.1394
	(0.5220)	(0.5411)	(0.5687)	(0.6043)	(0.6228)	(0.5230)	(0.5416)	(0.5710)	(0.6024)	(0.6172)
Caucasian	0.3337	0.3013	−0.1976	−0.2696	−0.2065	0.3472	0.3071	−0.2440	−0.3170	−0.2441
	(0.4067)	(0.4142)	(0.4550)	(0.4566)	(0.4795)	(0.4087)	(0.4171)	(0.4621)	(0.4661)	(0.4829)
Age		−0.0113***	−0.0049	−0.0005	0.0058		−0.0116***	−0.0051	−0.0010	0.0059
		(0.0035)	(0.0068)	(0.0068)	(0.0073)		(0.0035)	(0.0068)	(0.0067)	(0.0073)
Education of father			1.3285***	0.6427***	0.5392**			1.3073***	0.6021***	0.5029**
			(0.1998)	(0.2246)	(0.2437)			(0.1988)	(0.2262)	(0.2452)
Education of mother				1.3202***	1.2882***				1.3224***	1.2935***
				(0.2566)	(0.2798)				(0.2589)	(0.2812)
Digit ratios of the right hand (DR)						1.5274	1.0130	−0.6515	−0.7196	−1.0845
						(1.5824)	(1.5943)	(2.1892)	(2.2667)	(2.2782)
Constant	−1.1537	−0.2438	−0.0711	−1.0610	−0.2952	−0.6767	0.4328	1.6028	1.4108	1.6496
	(1.5185)	(1.5582)	(2.0021)	(2.1264)	(2.1425)	(1.5798)	(1.6246)	(2.2141)	(2.2849)	(2.2860)

The third panel										
Digit ratios of the left hand (DL)	1.2933	0.3369	−0.8896	−1.2042	−1.3108					
	(1.4126)	(1.4461)	(1.8906)	(1.9660)	(1.9976)					
Asian	−0.5741	−0.7359	−0.3989	−0.3589	−0.0347	−0.5323	−0.7006	−0.3745	−0.3175	−0.0132
	(0.5427)	(0.5563)	(0.5580)	(0.6022)	(0.6074)	(0.5412)	(0.5570)	(0.5646)	(0.6158)	(0.6239)
Caucasian	0.3226	0.2733	−0.1388	−0.2053	−0.5635	0.3704	0.3076	−0.0953	−0.1497	−0.5165
	(0.3475)	(0.3564)	(0.4450)	(0.4545)	(0.4804)	(0.3430)	(0.3539)	(0.4426)	(0.4489)	(0.4763)
Age		−0.0244***	−0.0374***	−0.0360***	−0.0343***		−0.0241***	−0.0377***	−0.0364***	−0.0350***
		(0.0032)	(0.0062)	(0.0062)	(0.0065)		(0.0032)	(0.0062)	(0.0062)	(0.0065)
Education of father			1.5438***	1.0364***	0.9421***			1.5480***	1.0423***	0.9489***
			(0.1608)	(0.1838)	(0.1873)			(0.1606)	(0.1829)	(0.1866)
Education of mother				0.9642***	0.9658***				0.9673***	0.9718***
				(0.1931)	(0.2010)				(0.1928)	(0.2010)
Digit ratios of the right hand (DR)						3.0578**	2.0752	1.0620	1.5075	1.1076
						(1.4761)	(1.4823)	(2.0298)	(2.0917)	(2.1322)
Constant	−1.4935	0.6954	2.0437	2.2011	2.2661	−3.2550**	−1.0556	0.1024	−0.4994	−0.1383
	(1.4116)	(1.4706)	(1.9094)	(1.9921)	(2.0188)	(1.4748)	(1.5043)	(2.0500)	(2.1109)	(2.1431)
										
Log-likelihood	−2122.3269	−2042.7570	−1109.1569	−1080.0024	−1031.6749	−2119.3198	−2043.3170	−1109.3574	−1080.5485	−1031.6670
Log-likelihood, constant term only	−2125.4235	−2125.4235	−1199.0797	−1188.6772	−1170.3001	−2125.4235	−2125.4235	−1199.0797	−1188.6772	−1170.3001
Wald chi2	5.6738	207.3747	145.1196	162.2404	221.5683	11.8531	198.9310	145.6110	161.6998	220.5490
Prob > chi2	0.7721	0.0000	0.0000	0.0000	0.0000	0.2217	0.0000	0.0000	0.0000	0.0000
Pseudo R2	0.0015	0.0389	0.0750	0.0914	0.1185	0.0029	0.0386	0.0748	0.0910	0.1185
Number of observations	1700	1700	1039	1031	1016	1700	1700	1039	1031	1016

*Note*: Robust standard errors in parentheses. Specifications (5) and (10) include secondary school regional dummies, according to settlement types. Individuals which are not belong to “Asian” or “Caucasian” groups constitute the reference group. In some specifications for females STATA returns errors, outcomes with a predicted probability that is less than 0. Details available upon request.

* p<0.1,

** p<0.05,

*** p<0.01.

**Table 15:** Generalized logistic regression output, regression coefficients. Self-identities of nationalities are included. Dependent variable – educational attainment. Males. The subsample created by the first method of the deletion of outliers.

	(1)	(2)	(3)	(4)	(5)	(6)	(7)	(8)	(9)	(10)
The first panel										
Digit ratios of the left hand (DL)	7.1523**	7.1864**	6.2690*	5.6492	5.0875					
	(2.8042)	(2.8233)	(3.5870)	(3.5918)	(3.8211)					
Asian	13.6234***	11.9688***	13.7520***	13.2196***	13.3554***	13.4621***	12.6899***	12.0702***	13.1740***	13.3174***
	(0.3160)	(0.3585)	(0.4086)	(0.4473)	(0.5689)	(0.3203)	(0.3637)	(0.4020)	(0.4523)	(0.5717)
Caucasian	−0.8834*	−0.8714*	−1.3357***	−1.1993**	−1.3174**	−0.9552**	−0.9206*	−1.4316***	−1.3054**	−1.4267**
	(0.4713)	(0.4801)	(0.5022)	(0.5643)	(0.5867)	(0.4804)	(0.4892)	(0.5175)	(0.5890)	(0.5957)
Age		−0.0172*	0.0353***	0.0326**	0.0387**		−0.0164*	0.0376***	0.0346**	0.0405**
		(0.0091)	(0.0132)	(0.0137)	(0.0158)		(0.0092)	(0.0138)	(0.0144)	(0.0164)
Education of father			1.2508***	1.0810**	1.0578**			1.2717***	1.0989**	1.0821**
			(0.4534)	(0.5082)	(0.5341)			(0.4494)	(0.4974)	(0.5252)
Education of mother				0.2312	0.3857				0.2241	0.3854
				(0.4734)	(0.5081)				(0.4700)	(0.5065)
Digit ratios of the right hand (DR)						7.0582***	6.4361**	6.3295*	6.4183*	5.9393
						(2.6727)	(2.6569)	(3.5590)	(3.6798)	(3.8319)
Constant	−4.9232*	−4.1332	−5.3626	−4.6326	−4.2378	−4.8261*	−3.4257	−5.5166	−5.4764	−5.1527
	(2.7806)	(2.8004)	(3.6337)	(3.6572)	(3.8454)	(2.6506)	(2.6686)	(3.7449)	(3.8768)	(4.0112)

The second panel										
Digit ratios of the left hand (DL)	5.1175***	5.1184***	5.1722**	5.6908**	5.5759**					
	(1.7729)	(1.7720)	(2.2733)	(2.2850)	(2.3627)					
Asian	−0.3231	−0.2230	−0.1726	−0.4566	−0.4217	−0.3518	−0.2473	−0.2278	−0.5139	−0.4599
	(0.5719)	(0.5735)	(0.6783)	(0.7111)	(0.8941)	(0.5772)	(0.5787)	(0.6885)	(0.7231)	(0.9127)
Caucasian	0.0414	0.0220	−0.0874	0.0925	0.0145	0.0276	0.0117	−0.1224	0.0460	−0.0575
	(0.3883)	(0.3941)	(0.4030)	(0.4455)	(0.4702)	(0.4001)	(0.4067)	(0.4119)	(0.4591)	(0.4861)
Age		0.0072*	−0.0093	−0.0068	−0.0046		0.0074*	−0.0083	−0.0057	−0.0033
		(0.0043)	(0.0078)	(0.0079)	(0.0084)		(0.0043)	(0.0078)	(0.0079)	(0.0084)
Education of father			1.4745***	1.1644***	1.1044***			1.4856***	1.1819***	1.1327***
			(0.2101)	(0.2390)	(0.2395)			(0.2106)	(0.2407)	(0.2420)
Education of mother				0.5562**	0.5275**				0.5516**	0.5218**
				(0.2418)	(0.2481)				(0.2430)	(0.2515)
Digit ratios of the right hand (DR)						4.5405**	4.5776**	5.3687**	6.0948**	6.5447***
						(1.8191)	(1.8133)	(2.3437)	(2.4047)	(2.5217)
Constant	−4.7121***	−5.0474***	−4.7559**	−5.4009**	−5.2151**	−4.1352**	−4.5156**	−4.9956**	−5.8477**	−6.2298**
	(1.7680)	(1.7780)	(2.2716)	(2.2826)	(2.3618)	(1.8135)	(1.8175)	(2.3694)	(2.4241)	(2.5339)

The third panel										
Digit ratios of the left hand (DL)	5.5751***	5.6063***	4.7359**	5.1822**	4.4299*					
	(1.7886)	(1.7925)	(2.1973)	(2.2078)	(2.2998)					
Asian	0.2154	0.3603	0.4765	0.2366	0.5487	0.2036	0.3482	0.5180	0.2697	0.5702
	(0.5646)	(0.5648)	(0.6335)	(0.6142)	(0.7361)	(0.5692)	(0.5696)	(0.6359)	(0.6216)	(0.7726)
Caucasian	−0.3002	−0.3087	−0.4232	−0.3194	−1.0155	−0.2875	−0.2956	−0.3977	−0.3028	−1.0175*
	(0.4014)	(0.3988)	(0.5363)	(0.5943)	(0.6214)	(0.4017)	(0.4003)	(0.5264)	(0.5832)	(0.6095)
Age		0.0098**	−0.0036	0.0008	−0.0003		0.0098**	−0.0037	0.0008	−0.0004
		(0.0041)	(0.0082)	(0.0084)	(0.0087)		(0.0041)	(0.0083)	(0.0085)	(0.0087)
Education of father			1.5814***	1.0788***	1.0926***			1.5987***	1.0943***	1.1123***
			(0.1832)	(0.2144)	(0.2273)			(0.1840)	(0.2189)	(0.2329)
Education of mother				0.9350***	0.8641***				0.9421***	0.8690***
				(0.2237)	(0.2368)				(0.2276)	(0.2422)
Digit ratios of the right hand (DR)						5.3527***	5.3642***	7.4552***	7.9905***	7.6220***
						(1.8761)	(1.8743)	(2.3424)	(2.3680)	(2.5840)
Constant	−6.0384***	−6.5300***	−5.5697**	−6.2769***	−5.3185**	−5.8144***	−6.2883***	−8.2901***	−9.0864***	−8.5027***
	(1.7878)	(1.8027)	(2.2042)	(2.2130)	(2.3166)	(1.8743)	(1.8810)	(2.3839)	(2.3992)	(2.6163)
										
Log-likelihood	−1282.3784	−1274.6914	−804.0535	−779.7735	−741.3115	−1283.2537	−1275.9066	−802.3743	−777.9081	−739.3694
Log-likelihood, constant term only	−1293.6849	−1293.6849	−862.7502	−843.9370	−831.6926	−1293.6849	−1293.6849	−862.7502	−843.9370	−831.6926
Wald chi2	1938.4443	1522.2746	1343.1041	1277.3790	3267.2616	1829.6738	1641.0251	1082.6532	1313.8004	3241.3021
Prob > chi2	0.0000	0.0000	0.0000	0.0000	0.0000	0.0000	0.0000	0.0000	0.0000	0.0000
Pseudo R2	0.0087	0.0147	0.0680	0.0760	0.1087	0.0081	0.0137	0.0700	0.0782	0.1110
Number of observations	1001	1001	682	669	661	1001	1001	682	669	661

*Note*: Robust standard errors in parentheses. Specifications (5) and (10) include secondary school regional dummies, according to settlement types. Individuals which are not belong to “Asian” or “Caucasian” groups constitute the reference group.

* p<0.1,

** p<0.05,

*** p<0.01.

**Table 16:** Generalized logistic regression output, regression coefficients. Self-identities of nationalities are included. Dependent variable – educational attainment. Females. The subsample created by the second method of the deletion of outliers.

	(1)	(2)	(3)	(4)	(5)	(6)	(7)	(8)	(9)	(10)
The first panel										
Digit ratios of the left hand (DL)	3.6479**	0.5536	0.6778	0.4110	0.4098					
	(1.5274)	(1.5689)	(2.4601)	(2.5191)	(2.5738)					
Asian	0.0527	−0.9837	−0.8884	−1.0074	−1.1754	0.1260	−0.9986	−0.9239	−1.0737	−1.2308
	(0.7444)	(0.7849)	(0.7700)	(0.7700)	(0.8589)	(0.7457)	(0.7842)	(0.7862)	(0.7888)	(0.8726)
Caucasian	0.5452	−0.1005	−0.6590	−0.6607	−0.9326	0.5522	−0.0998	−0.6930	−0.7058	−0.9719
	(0.5989)	(0.6176)	(0.6235)	(0.6166)	(0.6572)	(0.6056)	(0.6210)	(0.6333)	(0.6259)	(0.6634)
Age		−0.0497***	0.0031	0.0055	0.0065		−0.0497***	0.0026	0.0048	0.0059
		(0.0041)	(0.0138)	(0.0144)	(0.0143)		(0.0041)	(0.0141)	(0.0147)	(0.0146)
Education of father			0.9588**	0.0869	−0.0363			0.9543**	0.0795	−0.0420
			(0.3800)	(0.4296)	(0.4715)			(0.3812)	(0.4281)	(0.4701)
Education of mother				1.9326***	2.3501***				1.9319***	2.3451***
				(0.6703)	(0.8314)				(0.6674)	(0.8285)
Digit ratios of the right hand (DR)						2.8084*	0.1024	−1.0696	−1.6133	−1.4092
						(1.6112)	(1.3420)	(2.9688)	(3.0822)	(3.0427)
Constant	−1.4450	4.4830***	2.1187	2.2257	2.2785	−0.6143	4.9364***	3.8932	4.2927	4.1313
	(1.5161)	(1.6347)	(2.7031)	(2.7729)	(2.8020)	(1.6040)	(1.4056)	(3.2462)	(3.3586)	(3.3032)

The second panel										
Digit ratios of the left hand (DL)	1.4872	1.0829	−0.2873	−0.1445	−0.4227					
	(1.0542)	(1.0582)	(1.3572)	(1.3945)	(1.4361)					
Asian	−0.2502	−0.4086	−0.3672	−0.3709	−0.1132	−0.2619	−0.4364	−0.4053	−0.4154	−0.1551
	(0.4579)	(0.4778)	(0.4796)	(0.4948)	(0.5288)	(0.4532)	(0.4731)	(0.4853)	(0.4973)	(0.5313)
Caucasian	0.2092	0.1513	−0.1860	−0.1907	−0.3077	0.2088	0.1400	−0.2135	−0.2170	−0.3298
	(0.3276)	(0.3400)	(0.3828)	(0.3846)	(0.3956)	(0.3301)	(0.3430)	(0.3851)	(0.3866)	(0.3965)
Age		−0.0128***	−0.0067	−0.0023	0.0011		−0.0134***	−0.0069	−0.0025	0.0009
		(0.0030)	(0.0058)	(0.0059)	(0.0062)		(0.0030)	(0.0059)	(0.0059)	(0.0062)
Education of father			1.3888***	0.7524***	0.6910***			1.3864***	0.7426***	0.6832***
			(0.1759)	(0.2148)	(0.2276)			(0.1760)	(0.2135)	(0.2261)
Education of mother				1.2410***	1.2462***				1.2475***	1.2499***
				(0.2444)	(0.2623)				(0.2438)	(0.2617)
Digit ratios of the right hand (DR)						0.3036	−0.3404	−1.1884	−1.1458	−1.1834
						(1.0618)	(1.0362)	(1.3517)	(1.3946)	(1.3999)
Constant	−0.7299	0.3370	1.2004	0.7882	1.0092	0.4510	1.7854*	2.1170	1.8056	1.7841
	(1.0520)	(1.0827)	(1.3948)	(1.4340)	(1.4748)	(1.0621)	(1.0700)	(1.4003)	(1.4413)	(1.4425)

The third panel										
Digit ratios of the left hand (DL)	1.1324	0.5291	−0.9077	−0.8723	−0.9857					
	(0.9998)	(1.0241)	(1.3479)	(1.3685)	(1.4023)					
Asian	−0.3786	−0.5667	−0.3970	−0.3171	−0.2368	−0.3381	−0.5561	−0.4179	−0.3313	−0.2371
	(0.4657)	(0.4664)	(0.4837)	(0.5103)	(0.5252)	(0.4648)	(0.4662)	(0.4874)	(0.5130)	(0.5252)
Caucasian	0.0877	0.0240	−0.2303	−0.2669	−0.6146	0.1096	0.0315	−0.2507	−0.2826	−0.6219
	(0.2971)	(0.3071)	(0.3695)	(0.3758)	(0.3931)	(0.2968)	(0.3077)	(0.3702)	(0.3763)	(0.3929)
Age		−0.0251***	−0.0409***	−0.0389***	−0.0375***		−0.0251***	−0.0412***	−0.0391***	−0.0377***
		(0.0029)	(0.0055)	(0.0055)	(0.0057)		(0.0029)	(0.0055)	(0.0056)	(0.0057)
Education of father			1.4887***	1.0035***	0.9254***			1.4936***	1.0095***	0.9307***
			(0.1405)	(0.1620)	(0.1637)			(0.1407)	(0.1619)	(0.1634)
Education of mother				0.9598***	0.9509***				0.9586***	0.9498***
				(0.1731)	(0.1768)				(0.1728)	(0.1765)
Digit ratios of the right hand (DR)						1.5866	0.4723	−1.1730	−0.9724	−0.7865
						(1.0102)	(1.0116)	(1.3417)	(1.3607)	(1.3906)
Constant	−1.4314	0.4405	2.1111	1.8927	1.9484	−1.8867*	0.4980	2.3879*	2.0018	1.7556
	(0.9994)	(1.0446)	(1.3834)	(1.4071)	(1.4381)	(1.0108)	(1.0393)	(1.3866)	(1.4074)	(1.4357)
										
Log−likelihood	−2682.4111	−2575.0949	−1409.7966	−1373.2650	−1327.1711	−2681.9169	−2575.2053	−1409.6465	−1373.1288	−1327.0810
Log-likelihood, constant term only	−2686.3630	−2686.3630	−1522.8522	−1507.8976	−1487.4098	−2686.3630	−2686.3630	−1522.8522	−1507.8976	−1487.4098
Wald chi2	7.8176	274.2575	186.1425	203.3881	251.3254	8.3491	276.5028	186.1338	202.4382	250.7580
Prob > chi2	0.5526	0.0000	0.0000	0.0000	0.0000	0.4994	0.0000	0.0000	0.0000	0.0000
Pseudo R2	0.0015	0.0414	0.0742	0.0893	0.1077	0.0017	0.0414	0.0743	0.0894	0.1078
Number of observations	2106	2106	1293	1283	1266	2106	2106	1293	1283	1266

*Note*: Robust standard errors in parentheses. Specifications (5) and (10) include secondary school regional dummies, according to settlement types. Individuals which are not belong to “Asian” or “Caucasian” groups constitute the reference group. In some specifications for females STATA returns errors, outcomes with a predicted probability that is less than 0. Details available upon request.

* p<0.1,

** p<0.05,

*** p<0.01.

**Table 17:** Generalized logistic regression output, regression coefficients. Self-identities of nationalities are included. Dependent variable – educational attainment. Males. The subsample created by the second method of the deletion of outliers.

	(1)	(2)	(3)	(4)	(5)	(6)	(7)	(8)	(9)	(10)
The first panel										
Digit ratios of the left hand (DL)	3.9374**	3.8984**	4.7261**	4.7195**	4.8528**					
	(1.7691)	(1.7771)	(2.1743)	(2.2057)	(2.2731)					
Asian	14.4307***	12.6778***	13.2933***	12.3300***	14.7755***	14.3995***	12.8950***	13.3824***	13.4129***	14.7293***
	(0.2765)	(0.3113)	(0.3557)	(0.3940)	(0.5089)	(0.2769)	(0.3122)	(0.3513)	(0.3892)	(0.5081)
Caucasian	−0.3309	−0.3343	−0.7427	−0.5874	−0.7193	−0.3746	−0.3756	−0.8044	−0.6646	−0.7870
	(0.4544)	(0.4582)	(0.4918)	(0.5556)	(0.5814)	(0.4527)	(0.4551)	(0.4945)	(0.5605)	(0.5739)
Age		−0.0153**	0.0260**	0.0271**	0.0312**		−0.0153**	0.0261**	0.0271**	0.0311**
		(0.0076)	(0.0117)	(0.0121)	(0.0139)		(0.0076)	(0.0117)	(0.0121)	(0.0137)
Education of father			1.0051***	0.6178	0.5899			1.0107***	0.6305*	0.6059
			(0.3414)	(0.3778)	(0.4001)			(0.3411)	(0.3759)	(0.3969)
Education of mother				0.6687	0.6399				0.6531	0.6315
				(0.4086)	(0.4259)				(0.4051)	(0.4208)
Digit ratios of the right hand (DR)						2.6624	2.4905	3.2793	3.3296	3.1010
						(1.9439)	(1.8941)	(2.6342)	(2.7106)	(2.7233)
Constant	−1.8011	−1.0372	−3.5596	−3.6177	−3.8972	−0.5391	0.3589	−2.1362	−2.2422	−2.1524
	(1.7531)	(1.8297)	(2.2716)	(2.3257)	(2.3983)	(1.9294)	(1.9337)	(2.7527)	(2.8381)	(2.8476)

The second panel										
Digit ratios of the left hand (DL)	2.7835**	2.9088**	3.5978**	3.8290**	3.5805**					
	(1.2300)	(1.2295)	(1.5602)	(1.5887)	(1.6405)					
Asian	0.0825	0.1673	0.0355	−0.1459	0.1262	0.0548	0.1356	0.0256	−0.1635	0.1198
	(0.5254)	(0.5257)	(0.6206)	(0.6538)	(0.7849)	(0.5298)	(0.5317)	(0.6390)	(0.6753)	(0.8137)
Caucasian	0.2019	0.1942	0.0481	0.2768	−0.0610	0.1796	0.1723	−0.0073	0.2052	−0.1512
	(0.3302)	(0.3322)	(0.3464)	(0.3811)	(0.4232)	(0.3297)	(0.3314)	(0.3394)	(0.3735)	(0.4189)
Age		0.0071*	−0.0109	−0.0083	−0.0043		0.0068*	−0.0109	−0.0083	−0.0042
		(0.0038)	(0.0069)	(0.0070)	(0.0074)		(0.0038)	(0.0069)	(0.0070)	(0.0074)
Education of father			1.5008***	1.1300***	1.0609***			1.5006***	1.1312***	1.0653***
			(0.1816)	(0.1998)	(0.2058)			(0.1822)	(0.2006)	(0.2068)
Education of mother				0.6850***	0.6152***				0.6845***	0.6242***
				(0.2029)	(0.2127)				(0.2038)	(0.2130)
Digit ratios of the right hand (DR)						1.3825	1.4507	2.7030*	2.9438*	2.8791*
						(1.2761)	(1.2773)	(1.6072)	(1.6328)	(1.6827)
Constant	−2.4168**	−2.8686**	−3.1716**	−3.5496**	−3.3802**	−1.0236	−1.4077	−2.2809	−2.6692	−2.6844
	(1.2245)	(1.2451)	(1.5805)	(1.6086)	(1.6637)	(1.2714)	(1.2924)	(1.6443)	(1.6631)	(1.7082)

The third panel										
Digit ratios of the left hand (DL)	3.6033***	3.8118***	3.7738**	3.8872**	3.1692*					
	(1.2682)	(1.2690)	(1.6164)	(1.6512)	(1.7366)					
Asian	0.0921	0.2280	−0.0059	−0.1117	0.0753	0.1026	0.2344	0.0529	−0.0543	0.1063
	(0.5174)	(0.5088)	(0.6623)	(0.5718)	(0.6350)	(0.5121)	(0.5060)	(0.6736)	(0.5827)	(0.6552)
Caucasian	−0.0893	−0.0894	−0.1792	−0.0154	−0.6953	−0.1221	−0.1233	−0.2448	−0.0898	−0.7698
	(0.3222)	(0.3187)	(0.4320)	(0.4556)	(0.5209)	(0.3287)	(0.3256)	(0.4320)	(0.4586)	(0.5263)
Age		0.0106***	−0.0053	−0.0011	−0.0003		0.0103***	−0.0054	−0.0013	−0.0006
		(0.0036)	(0.0072)	(0.0073)	(0.0075)		(0.0036)	(0.0072)	(0.0074)	(0.0075)
Education of father			1.6482***	1.1269***	1.0895***			1.6597***	1.1336***	1.0995***
			(0.1599)	(0.1821)	(0.1899)			(0.1603)	(0.1833)	(0.1917)
Education of mother				1.0201***	0.9701***				1.0296***	0.9783***
				(0.1895)	(0.1990)				(0.1909)	(0.2005)
Digit ratios of the right hand (DR)						2.5407*	2.6675**	4.2749***	4.5372***	3.8330**
						(1.3047)	(1.2994)	(1.6358)	(1.6308)	(1.7072)
Constant	−4.0506***	−4.7529***	−4.5574***	−4.9448***	−4.1824**	−2.9922**	−3.5993***	−5.0568***	−5.5885***	−4.8331***
	(1.2656)	(1.2858)	(1.6516)	(1.6883)	(1.7796)	(1.3023)	(1.3142)	(1.7005)	(1.6919)	(1.7670)
										
Log-likelihood	−1696.9798	−1687.0315	−1070.0935	−1034.8209	−994.7221	−1699.3774	−1689.7042	−1070.4478	−1035.0376	−995.2256
Log-likelihood, constant term only	−1704.9478	−1704.9478	−1145.2661	−1120.8928	−1105.4140	−1704.9478	−1704.9478	−1145.2661	−1120.8928	−1105.4140
Wald chi2	2765.1351	2164.2124	1627.1806	1370.7267	5243.5736	2756.5703	2222.7958	1691.3757	1729.6323	5324.7872
Prob > chi2	0.0000	0.0000	0.0000	0.0000	0.0000	0.0000	0.0000	0.0000	0.0000	0.0000
Pseudo R2	0.0047	0.0105	0.0656	0.0768	0.1001	0.0033	0.0089	0.0653	0.0766	0.0997
Number of observations	1318	1318	901	884	873	1318	1318	901	884	873

*Note*: Robust standard errors in parentheses. Specifications (5) and (10) include secondary school regional dummies, according to settlement types. Individuals which are not belong to “Asian” or “Caucasian” groups constitute the reference group.

* p<0.1,

** p<0.05,

*** p<0.01.
